# The social and health implications of digital work intensification. Associations between exposure to information and communication technologies, health and work ability in different socio-economic strata

**DOI:** 10.1007/s00420-020-01588-5

**Published:** 2020-10-21

**Authors:** Prem Borle, Franziska Boerner-Zobel, Susanne Voelter-Mahlknecht, Hans Martin Hasselhorn, Melanie Ebener

**Affiliations:** 1 Charité - Universitätsmedizin Berlin, corporate member of Freie Universität Berlin, Humboldt-Universität zu Berlin, and Berlin Institute of Health, Institute of Occupational Medicine, Augustenburger Platz 1, 13353 Berlin, Germany; 2 Department of Occupational Health Science, University of Wuppertal, Wuppertal, Germany

**Keywords:** Technostress, Digital divide, Ageing workers, Job requirement level, Occupational status, Workplace well-being

## Abstract

**Purpose:**

Older employees are often thought to be vulnerable to negative effects of information and communication technology (ICT). Our study aims to examine associations between work-related ICT exposure (i.e. ICT use or digital work intensification), physical health, mental health and work ability (WA). We examine whether these associations are modified by socio-economic position (SEP).

**Methods:**

We analysed cross-sectional data from 3180 participants (born in 1959 and 1965) in wave 3 of the representative German lidA cohort study. We performed hierarchical multiple regression to assess the distinct associations of ICT use and digital work intensification with mental and physical health and WA. We stratified analyses by SEP and controlled for age, sex, and digital affinity.

**Results:**

92% of participants reported ICT use at work. Almost 20% reported high levels of digital work intensification, while a similar proportion did not experience digital work intensification. In bivariate analyses, ICT use by itself was not significantly associated with mental health or WA in the total sample or when stratified. Digital work intensification displayed negative associations with mental health and WA. In hierarchical multiple regressions, digital work intensification showed consistently negative associations with mental health and work ability of similar strength across SEP.

**Conclusion:**

Our results suggest that ICT use, per se, does not negatively impact older workers. Digital work intensification may be associated with worse mental health and work ability. Research on health and social implications of work-related ICT should differentiate patterns of ICT exposure and assess modifications by SEP to better gauge the ambiguous effects of ICT.

## Introduction

### Health implications of work-related exposure to ICT

The increasing diffusion of information and communications technologies (ICT), defined as technologies that provide access to information through telecommunications, has been transforming work and life in the twenty-first century. ICT use at work has intensified with the use of ever more devices including laptops, tablets, smartphones and, more recently, wearables like smart watches and data glasses. Data, multimedia content, social networks and computer‐processing power can be accessed from almost anywhere, creating what has been described as an “omnipresent connectivity” (Holtgrewe 2014), which allows some forms of work to be performed anywhere and at any time. However, an action commonly framed as evidence of personal autonomy—choosing to use one’s mobile email devices to work anywhere/anytime—can lead professionals to feel like they are obliged to do it everywhere/all the time, thus actually diminishing their autonomy in practice. This ambiguous phenomenon has been called “the autonomy paradox” (Mazmanian et al. 2013). ICT influence information processing at work and have become increasingly ubiquitous with often ambiguous effects on many aspects of workers’ health. Thus, it is crucial to assess the rapid diffusion of work-related ICT use and the ongoing transformations of work and private life (Wajcman 2016) with regard to their implications for health.

Digitalisation—often conceptualised in broad strokes—has been repeatedly hyped as either a problem or a solution for work life in political and economic discussions. From the perspective of the users, however, exposure to ICT can require high physical, social, and cognitive skills (Ayyagari et al. 2011), potentially leading users to experience stress when using ICT (Ragu-Nathan et al. 2008; Tarafdar et al. 2007), and hence to perceive stress as a downside of using ICT. Previous studies have shown associations between ICT demands and cognitive disturbances (Stenfors et al. 2013), suboptimal self-rated health (Stadin et al. 2016, 2019), and associations between ICT use and burnout (Berg-Beckhoff et al. 2017; Silva et al. 2016). To describe the health implications of ICT use at work, the term “technostress” has gained increasing popularity in various disciplines, such as Information Systems and psychology. The term was first coined by Brod (1982) to describe an increasing imbalance between people and computers, that is, a lack of adaption from a psychological perspective. In this paper, we use the more general and neutral term ICT exposure to encompass what previous studies have called technostress, digital stress or ICT demands.

Higher job demands have been described as not necessarily bad for workers’ health in some cases when the job demands are balanced with adequate resources (Bakker and Demerouti 2007; Karasek and Theorell 1990). Many studies thus emphasise the role of resources to mitigate potentially negative effects, such as shown in discussions about the impact of both social support and ICT-related support (Day et al. 2012; Ragu-Nathan et al. 2008). Lazarus’ transaction theory of stress from organisational psychology (Lazarus 1966) draws attention to the conditions under which ICT exposure is experienced negatively. It has been used as a basis to study the health implications of ICT exposure within the model of technostress (Tarafdar et al. 2015) and emphasises that stress can result from a combination of a demand condition that causes the stress (stress creators or ‘stressors’) and the individual’s response to it (manifest adverse outcomes referred to as ‘strain’). By shaping the working conditions that frame an individual’s response, organisational factors may influence whether ICT use is perceived as strain. An EU foresight study (EU-OSHA 2018) suggests that both psychosocial and organisational factors will become more important for occupational health as digitalising work can drive changes that can increase the risk of workers’ stress (e.g. increased monitoring of workers, an assumption of 24/7 availability and the management of work and workers by algorithms).

A better understanding of the conditions under which ICT use represents an opportunity or a concern and whether or not it has negative effects on health requires further exploration. Although extremely varied forms of ICT use exist in the working population, studies examining occupational ICT exposure have often been limited to highly-qualified occupations. A recent systematic review emphasised the importance of meaningful distinctions between occupational characteristics and found that only few studies sufficiently specified organisational factors influencing ICT-related work processes (Berg-Beckhoff et al. 2017). Depending on organisational factors, ICT exposure may increase working hours, create interference between work and personal life, or result in work intensification (Eurofound and ILO 2017). Work intensification has been an influential trend from a historical perspective (Green 2004) and continues to shape the effects of ICT use at work in the form of what can be seen as digital work intensification (Carayon 2007; Meyer et al. 2019). A study by Chesley (2014) aimed to assess the pathways through which ICT use may influence levels of employee strain and distress and found that use is linked to higher levels of employee strain and distress via a work intensification process. Thus, to assess whether the implications of ICT use for health depend on broader organisational factors, we distinguish between ICT use and digital work intensification.

### Social implications of work-related exposure to ICT

#### The role of socioeconomic position

Researchers of ICT use in the context of leisure as opposed to work have called for closer attention to the relationship between ICT use on the one hand, and social inequalities on the other. When examining social implications of digitalisation or digital divides—i.e. inequalities linked to digitalisation—commonly used dimensions to describe the divide include socio-economic data, age and geographic distribution. Research typically examines who, with which characteristics, connects how, to what when it comes to ICT use (Hilbert 2011). Some scholars have suggested going beyond the familiar idea of the ‘digital divide’ to develop a focus on digital social inequality (Halford and Savage 2010), while others emphasise how the impact of digital inequality continues to expand in many directions, including health (Weiss et al. 2018). Inequalities due to varying working conditions impact workers’ health tremendously (Scheil-Adlung and Sandner 2010). Yet, the relationship between digital inequalities and other forms of inequality, such as occupational health inequalities, are still poorly understood (Robinson et al. 2015).

The effects of work-related ICT exposure on health may differ by socioeconomic position (SEP). It should be noted that a wide range of different variables have been used to study SEP, such as income, education, job requirement level and having a managerial position. Previous research has shown that with higher levels of education and SEP, levels of ICT use increase (Arnold et al. 2017; Stadin et al. 2016). A large-scale study in Sweden found a higher frequency of high ICT demands at work among people with intermediate and high SEP (Stadin et al. 2016). However, in the longitudinal analyses of associations between repeated exposures to high ICT demands at work and consecutive self-rated health, no statistically significant differences were found between participants with high and low SEP. The study could not fully explain this unexpected finding, reporting possible limitations in the scale used to measure ICT demands at work when not studying highly-qualified occupations (Stadin et al. 2019).

In their systematic review, Berg-Beckhoff et al. (2017) conclude that a limitation of most studies is that they do not allow for separate views on specific occupations or specific ICT tasks. The nature of work-related ICT use depends on the occupations and types of work tasks, which, in turn, vary greatly according to the level of qualification and positions in a social hierarchy (Berg-Beckhoff et al. 2017). A case in point is the increasing interest in new forms of digital labour and precarious work from policy-makers, governments and research organisations, for instance with regards to click-workers or the gig economy (Graham et al. 2017). Thus, our study also explores the usefulness of the stratification variable “job requirement level” as a way of addressing both the importance of distinguishing SEP and occupational tasks in the context of digitalised work.

#### The role of age

In many countries, older workers are the fastest growing segment of ICT users, yet they are often included in studies of ICT-related stress only as minority users (Nimrod 2018). It is a popular belief that, due to lacking skills resulting from a digital divide by age, older generations are more susceptible to the negative effects of ICT (O'Driscoll et al. 2010). But previous research on the relationship between ICT, age and work-related health has been inconclusive and points to inconsistent implications for older workers.

Regarding the level of ICT exposure, some studies detected higher levels of ICT exposure among older workers (Marchiori et al. 2019), whereas the oft-cited studies by Ragu-Nathan et al. (2008) and Tarafdar et al. (2011) found evidence of more ICT exposure among younger workers. With regard to the implications of ICT exposure for physical health, there is a widely held belief that older workers, in particular, may benefit from a reduction of physical strain. Industry leaders and politicians have often echoed such hopes in response to demographic change. But the need to continuously improve their IT skills may also create challenges for older workers (Bellmann 2017; Hauke et al. 2020). Despite the widespread hopes that ICT may contribute to reducing physical strain, relatively little is known about the associations of digital work with physical health.

Other research has also examined the implications of ICT exposure for mental health and the role of age. A study by Shu et al. (2011) found that levels of ICT exposure increased with age. Another recent study on the associations between individual characteristics and ICT exposure found that older workers reported greater difficulties with the increase of technological complexity (Marchiori et al. 2019). In contrast, a longitudinal study challenges age stereotypes by revealing no age effects on the level of overall ICT exposure (Hauk et al. 2019). This study even found that older workers reported a lower level of ICT-related strain and, therefore, concluded that, in occupational settings, higher age is not connected to an increase of situations in which ICT-related demands exceed older workers’ abilities to meet them.

A systematic review of associations between ICT use and the health outcomes stress and burnout identified that ICT exposure was more prevalent in middle-aged workers (Berg-Beckhoff et al. 2017). Although until 2017, no study had investigated age as an effect modifier, previous research results suggest that older employees do not experience more work-related ICT exposure or burnout due to their use of ICT. This finding held, even when different outcome measurements for stress or burnout and exposure to several forms of ICT use were considered (Berg-Beckhoff et al. 2017). A recent German report (Gimpel et al. 2018) similarly found that ICT exposure was the highest among 25–34 and 35–44 year-olds. People aged 64 + reported the least ICT exposure, despite a decline in digital affinity by age, while high levels of ICT exposure were associated with worse health outcomes. Two further German studies found only weak associations of ICT exposure with health outcomes, such as emotional distress (Böhm 2016; Richter et al. 2017). In sum, previous research on mental health, mostly measured as stress or burnout, suggests that ICT exposure may be higher among the middle-aged working population and may decrease with age.

### Developing models for ICT exposure and health

Tarafdar et al. (2015) have acknowledged the importance of more conceptual development to better account for the role of context with current models of technostress. As suggested in a methodological paper (Nimrod 2018), models of ICT exposure could be improved if they differentiated relevant factors outside of work (e.g. privacy and complexity) versus at the workplace (e.g. work overload and invasion). Moreover, Hauk et al. (2019) have recommended investigating individual factors of ICT exposure with attention to the role of age. A nationally representative study in Germany found that two-thirds of employees (in companies with at least 50 employees) who used some form of ICT at work reported digital work intensification (BMAS 2016). The greater relative importance of work overload due to ICT as a central factor has been identified in a systematic review of technostress literature (La Torre et al. 2019) as well as individual studies (Gimpel et al. 2018; see also the validation study by Salanova et al. 2013). Overall, items related to digital work intensification have consistently shown higher reliability and associations with health outcomes (Day et al. 2012; Ragu-Nathan et al. 2008), therefore we focus on digital work intensification.

The most widely used models to study the effects of ICT use have been technostress (Ragu-Nathan et al. 2008) and ICT demands (Day et al. 2012). However, an issue with the term technostress is that it quickly presumes negative implications. As it tends to conflate multiple potential outcomes of ICT use, of which digital work intensification is just one, we opt for other terms in our study. The focus of our study is on work-related ICT exposure to understand its implications for occupational health. In examining work-related ICT exposure and how broader organisational factors influence the implications of ICT use for health, we distinguish between ICT use and digital work intensification.

This distinction guides our main research question: In what ways is ICT exposure (distinguishing the two aspects *intensity of ICT use* and *digital work intensification*) associated with general physical and mental health as well as work ability (WA) among older workers? In addition, our study aims to examine whether these associations are modified by socio-economic position (SEP). In what follows, we first describe the distribution of ICT exposure and then explore the individual contributions of its two interlinked aspects to the outcomes. All analyses also examine differences according to SEP.

## Methods

### Study sample

We used data from the third wave of the German lidA (an acronym for ‘living at work’) cohort study on work, age and health. The population sample of the lidA study consists of two cohorts of participants born either in 1959 or 1965 and in employment (i.e. subject to social security contributions on 31.12.2009). The initial study sample was selected from the ‘Integrated Employment Biographies’ (IEB) dataset, which includes all employees registered in Germany’s social security system (more than 80% of the German workforce). Informed consent was obtained from all individual participants included in the study. Trained professional interviewers collected data through computer-assisted personal interviews (CAPI) in the participants’ homes in 2011 and again in 2014 and 2018. The first wave reached a response rate of 27.3% and the third wave reached a follow-up response rate of 71.1%. The lidA Study received ethical approval and a subsequent renewal from the Ethics Commission of the University of Wuppertal on December 5th, 2008 and November 20th, 2017 [MS/BB 171,025 Hasselhorn].

For the present study, we analysed cross-sectional data from wave 3 (2018). We included participants that were employed for at least one hour per week (excluding people receiving old-age pension and the self-employed), resulting in a total sample of 3180. The data for stratification could only be obtained for *n* = 3133, so in the *stratified* analyses, this number is referred to as 100.0%. The sample is representative for the socially insured working population in Germany of the same ages, but excludes sworn civil servants and the self-employed. As part of the ‘baby boomer’ generations, it represents a considerable part of the current German workforce, which has been subject to new national policies aimed at increasing the legal retirement age (Hasselhorn et al. 2014). A detailed description of this study sample, including information on professions, has been published separately (Hasselhorn et al. 2014).

### Measures

#### ICT exposure: ICT use and digital work intensification

The *intensity of ICT* *use* was measured by nine questions as to the frequency of work-related use of specific ICT (incl. PC, laptop, mobile phone, email, websites etc.). The response options “other digital technologies” (used by *n* = 556) and “smart glasses” (used by *n* = 28) were excluded from further analysis due to being unspecific or relatively rare, respectively. The questions were answered on a scale of 0 (never) to 4 (very often). ICT use was computed as a mean score with 0 indicating low and 4 indicating high ICT use.

The variable *digital work intensification* measured the participants’ agreement on a Likert-like scale from 1 = ‘strongly disagree’ to 5 = ‘strongly agree’ with three items: (1) “Due to digital technologies at the workplace, I have more work than before.” (2) “The use of digital technologies increases my workload.” (3) “Due to digital technologies at the workplace, I must work faster than before.” We calculated a mean score and a high value (5) reflects high digital work intensification.

#### Physical and mental health

The lidA study assesses *self-rated physical and mental health* with twelve items based on the adapted and validated German version of the SF-12 Health Survey, a multipurpose short-form (SF) generic measure of health-related quality of life. The subscales used were: Physical Component Summary Scale (PCS) and Mental Component Summary Scale (MCS). The values were standardized by the recommended norm-based scoring and range from zero to 100 with higher values indicating better health and 50 corresponding to the average of the population in 2004 (Andersen et al. 2007; Gandek et al. 1998; Nübling et al. 2006; Ware et al. 2001, 1995).

#### Work ability

Work ability (WA) was measured with two items from the Work Ability Index. This short measure has been validated and proposed for the use in epidemiological studies (Ebener and Hasselhorn 2019). It consists of participants’ self-reported work ability regarding physical and mental work demands, each rated on one item with a five-point Likert-like scale from 1 = ‘very good’ to 5 = ‘very poor’. The resulting weighted sum score ranging from 2 to 10 (according to Tuomi et al. 1998) is then reversed. Thus, higher scores correspond with better WA.

#### Education

In accordance with recommendations from the German Society for Epidemiology, the information on highest school-leaving qualification was compared with the highest vocational training qualification and the combination was used to generate an eight-level ordinal index, which represents education in the sense of acquiring certificates that qualify for the pursuit of a professional activity. This index was trichotomised into three ordinal levels representing low/medium/high education.

#### Stratification by socioeconomic position

This study aimed to differentiate socioeconomic position (SEP), which was operationalised with the variable job requirement level. Given the focus on digitalised work, it appeared necessary to account for varying types of ICT use and ICT-related tasks, which may depend on both the complexity of tasks and occupational conditions. Job requirement level was generated as a variable reflecting the complexity of tasks and occupational conditions. During CAPI, the participants were asked in three steps to assign themselves according to the German classification scheme of occupations, ‘Klassifikation der Berufe’ 2010 (Paulus and Matthes 2013). The vertical dimension defines four levels of occupational qualifications depending on the skill levels generally required to perform these tasks and duties competently (see Table 1). As such, the skill level is a measure that is more closely related to the occupation than is the case with formal educational qualifications as the skills required to practice an occupation can also be acquired through work experience and on-the-job training (Hiesinger and Tophoven 2019). Therefore, unlike other measures of SEP, the variable gives priority to the degree of complexity of activities that is typical for a given occupation (Paulus and Matthes 2013).

**Table 1:** Four job requirement levels defined in KldB 2010 (Paulus and Matthes 2013)

Job requirement level		Normally required vocational qualification
1	Unskilled or semi-skilled activities	No vocational qualification, or regular one-year vocational training, required
2	Specialist activities	At least two years of vocational training, also graduation from vocational school
3	Complex specialist activities	Qualification as master craftsman or technician or equivalent technical school or college graduation, also graduation from a professional academy or university bachelor's degree
4	Highly complex activities	Completed university studies of at least four years

#### Control variables

Sex, age cohort (dichotomous) and digital affinity (dichotomous) were regarded as potential confounders. Digital affinity was operationalized based on the number of digital devices used privately (out of four). The cut-off was set between 2 or less (= low) and 3 or more devices (= high) as most participants used either a smartphone or a smartphone and a personal computer.

### Statistical analyses

All statistical analyses were conducted with IBM SPSS Statistics 25. Chi-square tests were conducted to analyse differences in proportions and ANOVAs were conducted to assess differences between the mean values of the four groups of job requirement level. Pearson´s r was calculated to determine correlations between ICT-related variables and health outcomes. Physical and mental health as well as Work Ability as outcomes were analysed separately with hierarchical regression models adjusted for age/cohort, sex and job requirement level. Alpha was set to 0.05, defining statistical significance. Analyses were carried out in the total study sample as well as stratified by job requirement level.

## Results

### Descriptives

#### Characteristics of participants

An overview of the main categorical characteristics in the sample is shown in Table 2. Chi-square tests revealed significant differences in distribution of all categorical characteristics between the subsamples of job requirement levels. The study population consisted of slightly more women (1770; 55.7%) than men (1406; 44.3%). Women are overrepresented in the lower job requirement levels (particularly unskilled and semi-killed: 75%) and men are especially overrepresented in occupations with highly complex tasks. The share of those born in 1965 (55.7%) was higher than the share of the older cohort born in 1959 (44.3%) which is due to deliberate oversampling of the younger cohort (Hasselhorn et al. 2014). With regard to job requirement level, a large majority of 1728 participants worked in specialist activities (55.2%), while only 224 (7.1%) participants worked in unskilled or semi-skilled activities. A total of 593 (18.9%) participants worked as complex specialists, and 588 (18.8%) worked in jobs categorized by highly complex activities. The levels of both educational status and digital affinity were distributed across the strata of job requirement levels as would be expected. For instance, among complex specialist and highly complex job requirement levels, half of the group showed high digital affinity, whereas among unskilled and semi-skilled only 23.7% did.

**Table 2:** Characteristics of the total study sample and stratified by job requirement level

	Total study sample *n* = 3180	Unskilled and semi-skilled *n* = 224 (7.1%^b^)	Specialist *n* = 1728 (55.2%)	Complex specialist *n* = 593 (18.9%)	Highly complex *n* = 588 (18.8%)	*p* value^a^
Cohort *n* (%)						< 0.01
1959	1406 (44.3)	122 (54.5)	748 (43.4)	266 (44.9)	246 (41.8)	
1965	1770 (55.7)	102 (45.5)	977 (56.6)	326 (55.1)	342 (58.2)	
Sex *n* (%)						< 0.001
Men	1438 (45.2)	56 (25.0)	702 (40.6)	305 (51.4)	350 (59.5)	
Women	1742 (54.8)	168 (75.0)	1026 (59.4)	288 (48.6)	238 (40.5)	
Education *n* (%)						< 0.001
Low	664 (21.0)	121 (54.8)	452 (26.4)	61 (10.4)	15 (2.6)	
Medium	1777 (56.3)	85 (38.5)	1146 (66.8)	392 (66.6)	132 (22.5)	
High	718 (22.7)	15 (6.8)	117 (6.8)	136 (23.1)	440 (75.0)	
Digital affinity *n* (%)						< 0.001
Low (0–2)	1883 (59.2)	171 (76.3)	1108 (64.1)	285 (48.1)	294 (50.0)	
High (3–4)	1297 (40.8)	53 (23.7)	620 (35.9)	308 (51.9)	294 (50.0)	

The column percentage is presented in parenthesis, e.g. there are 45.2% men and 54.8% women in the total study sample

*n* varies due to nonresponse

^a^Chi-square test for comparison of proportions between the subgroups

^b^Percentages of subgroups add up to 3133 as 100% (see “study sample”)

#### ICT exposure in the total sample and different job requirement levels

Overall, 8% reported no ICT use for work (i.e. an ICT use mean score of 0). In other words, 92% of respondents used at least one form of ICT for work-related purposes. The mean of ICT use was 1.53 in the total sample (SD: 0.92, see Table 3). The percentage of workers not experiencing any digital work intensification (i.e. a mean score of 0) was 21%, while about 18% experiences a high level of digital work intensification (i.e. mean score of 4 or higher). The average level of perceived digital work intensification in the total study sample was 2.58 (SD: 1.20).

**Table 3:** Characteristics in the total study sample and stratified by job requirement level (metric)

	Total study sample *n* = 3180 (103.6%) *n* = 3133 (100.0%)	Unskilled/semi-skilled *n* = 259 (7.5%)	Specialist *n* = 1907 (55.2%)	Complex specialist n = *653* (18.9%)	Highly complex *n* = 637 (18.4%)	* F*	*p value*^*a*^
ICT EXPOSURE							
ICT use (range 0–4) mean (SD)	1.53 (0.92)	0.39 (0.58)	1.29 (0.81)	1.96 (0.77)	2.25 (0.68)	315.56	< 0.001
Digital work intensification (range 1–5) mean (SD)	2.58 (1.20)	1.69 (1.05)	2.50 (1.21)	2.74 (1.15)	2.82 (1.13)	37.77	< 0.001
Outcomes							
Physical health (range 0–100) mean (SD)	48.16 (9.12)	44.13 (9.37)	46.96 (8.98)	49.76 (8.97)	51.52 (8.37)	49.16	< 0.001
Mental health (range 0–100) mean (SD)	51.68 (9.85)	50.69 (11.41)	51.65 (9.74)	52.22 (9.99)	51.61 (9.34)	0.64	n.s
Work ability (range 2–10) mean (SD)	7.78 (1.54)	7.25 (1.70)	7.66 (1.53)	8.03 (1.47)	8.05 (1.51)	18.31	< 0.001

*n* varies due to nonresponse

^a^ANOVA for comparisons of continuous variables

Both the proportions of ICT use and perceived digital work intensification differed significantly between job requirement level groups, i.e. increasing from lower to higher job requirement levels (see Table 3). Average ICT use was 1.53 (SD: 0.92) in the total sample and ranged from 0.39 (SD: 0.58) in the unskilled and semi-skilled to 2.25 (SD: 0.68) in highly complex job requirement levels. The average levels of perceived digital work intensification ranged from 1.69 (SD: 1.05) in the unskilled/semi-skilled to 2.82 (SD: 1.13) in the highly complex job requirement level.

#### Outcomes

Physical health and WA displayed statistically significant differences between job requirement level groups. Both increase with job requirement levels and thus correlate with higher SEP. In contrast, the differences in mental health were not significant, although the figures indicate a small, increasing trend from low to high job requirement level.

### Correlations

#### ICT use

When analysing the total study sample, the only relevant correlation found was the one between ICT use and physical health (Table 4; *r* = 0.21; *p* < 0.01). In the analyses stratified by job requirement level, the associations between ICT use and mental health as well as WA were of weak strength, falling below the correlation coefficient’s minimum 0.2-threshold for relevant associations (Ferguson 2009). In all analysed subsamples, associations of ICT use with mental health were not statistically significant.

**Table 4:** Pearson’s correlation coefficient between ICT use and health outcomes in the total sample and stratified by job requirement level

	Physical health	Mental health	Work ability
Unskilled/semi-skilled	0.07	0.01	0.06
Specialist	0.11**	0.00	0.09**
Complex specialist	0.10*	− 0.04	0.08
Highly complex	0.09*	− 0.02	0.07
*Total study sample*	0.21**	0.02	0.14**

N_total_ = 3180, N_strat_ = 3133

*p < 0.05

**p < 0.01

***p < 0.001

#### Digital work intensification

In the total study sample, digital work intensification showed a relevant negative association with mental health (Table 5; *r* = − 0.20; *p* < 0.01) and work ability (*r* = − 0.21; *p* < 0.01). When stratifying analyses by job requirement level, statistically significant negative associations were consistently found between digital work intensification and mental health in all groups. The strongest associations were to be found in the highly complex group (*r* = 0.26; *p* < 0.01). Similarly, WA displayed relevant negative associations in the three higher job requirement levels (*r* = 0.21–0.27; *p* < 0.01). Based on the analysis of crude correlations, there could be a potential concentration of negative outcomes in higher job requirement levels, which would need to be confirmed when controlling for confounders and ICT use.

**Table 5:** Pearson’s correlation coefficient between digital work intensification and health outcomes in the total sample and stratified by job requirement level

	Physical health	Mental health	Work ability
Unskilled/semi-skilled	0.04	− 0.20**	− 0.13
Specialist	0.02	− 0.18**	− 0.21**
Complex specialist	− 0.04	− 0.22**	− 0.27**
Highly complex	− 0.01	− 0.26**	− 0.27**
*Total study sample*	0.04*	− 0.20**	− 0.21**

N_total_ = 3180, N_strat_ = 3133

*p < 0.05

**p < 0.01

***p < 0.001

### Multiple regression analyses

In the regression analyses, effects of exposures on all outcomes were analysed for the total study sample and then additionally stratified by job requirement level, respectively (Table 6). Both ICT exposure variables were entered into the model together as they partially overlap (i.e. digital work intensification is conditional upon ICT use). In this way, the respective effects of ICT use and digital work intensification on health were examined while adjusting for each other. All analyses were controlled for the variables age cohort, sex and digital affinity. Following Ferguson’s (2009) recommendations for the interpretation of effect size estimates, we define *β* = 0.20 as the minimum threshold for a relevant strength of association. Associations below this threshold often reflect an over-interpretation of results because the quality of measurement and sampling strategies may inflate or attenuate the resultant effects.

**Table 6:** Multiple linear regressions

Job requirement level	Physical health			Mental health			Work ability		
	B	95% CI	*β*	B	95% CI	*β*	B	95% CI	*β*
Unskilled/semi-skilled									
ICT use	1.16	[− 2.22, 4.55]	0.08	1.33	[− 2.61, 5.26]	0.07	0.35	[− 0.20, 0.89]	0.14
Digital work intensification	0.25	[− 1.68, 2.17]	0.03	− 2.82**	[− 5.06, − 0.59]	− 0.26	− 0.35*	[− 0.66, − 0.04]	− 0.23
		*∆* *R*^*2*^ = 0.01			*∆ R*^*2*^ = 0.05			*∆ R*^*2*^ = 0.04	
		*n* = 138			*n* = 138			*n* = 139	
Specialist									
ICT use	1.46***	[0.80, 2.12]	0.12	1.19***	[0.49, 1.89]	0.09	0.40***	[0.29, 0.50]	0.20
Digital work intensification	− 0.26	[− 0.66, 0.14]	− 0.04	− 1.71***	[− 2.13, − 1.28]	− 0.21	− 0.38***	[− 0.44, − 0.32]	− 0.30
		*∆ R*^2^ = 0.01			*∆ R*^2^ = 0.04			*∆ R*^*2*^ = 0.08	
		*n* = 1661			*n* = 1661			*n* = 1664	
Complex specialist									
ICT use	0.69	[− 0.34, 1.71]	0.06	0.79	[− 0.33, 1.91]	0.06	0.21**	[0.05, 0.38]	0.11
Digital work intensification	− 0.52	[− 1.16, 0.12]	− 0.07	− 2.09***	[− 2.78, − 1.39]	− 0.24	− 0.40***	[− 0.50, − 0.29]	− 0.31
		*∆ R*^2^ = 0.01			*∆ R*^2^ = 0.06			*∆ R*^*2*^ = 0.09	
		*n* = 612			*n* = 612			*n* = 612	
Highly complex									
ICT use	1.01	[− 0.07, 2.09]	0.08	0.02	[− 1.14, 1.19]	0.00	0.19*	[0.01, 0.38]	0.09
Digital work intensification	− 0.20	[− 0.82, 0.41]	− 0.03	− 2.26***	[− 2.92, − 1.59]	− 0.27	− 0.39***	[− 0.50, − 0.28]	− 0.29
		*∆ R*^*2*^ = 0.01			*∆ R*^2^ = 0.07			*∆ R*^*2*^ = 0.08	
		*n* = 620			*n* = 620			*n* = 619	
*Total study sample*									
ICT use	0.99***	[0.79, 1.19]	0.19	0.47***	[0,25, 0.68]	0.08	0.19***	[0,16, 0.23]	0.22
Digital work intensification	− 0.17	[− 0.45, 0.11]	− 0.02	− 1.82***	[− 2,12, − 1,51]	− 0.22	− 0.35***	[− 0,40, − 0.31]	− 0.28
		*∆ R*^*2*^ = 0.03			*∆ R*^*2*^ = 0.05			*∆ R*^*2*^ = 0.09	
		*n* = 3078			*n* = 3078			*n* = 3081	

*B* unstandardised regression coefficient, *CI 95%* confidence interval, *β* standardised regressions coefficient, *∆ R*^*2*^ share of explained variance attributable to exposure variables, *n* varies due to an internal attrition; all analyses were controlled for year of birth, sex, and digital affinity

*p < 0.05

**p < 0.01

***p < 0.001

#### Effects of ICT exposure on physical health

In the total study sample, the weak positive association of ICT use with physical health (*β* = 0.19; *p* < 0.001) was no longer of relevant strength (Ferguson 2009). Digital work intensification showed no significant associations with physical health. When stratified, neither ICT use, nor digital work intensification had any significant associations with physical health in any subsample. For the total sample, the explained variance is 3%. Within each of the subsamples, ICT exposure explains only 1% variance in physical health.

#### Effects of ICT exposure on mental health

In the total study sample, ICT use showed no association of relevant strength with mental health. In contrast, digital work intensification showed a significant negative effect on mental health (*β* = − 0.22; *p* < 0.001). Furthermore, when stratified, there consistently remained significant negative effects of digital work intensification on mental health across all subsamples. The strongest effects were in the lowest (unskilled/semi-skilled employees: *β* = − 0.26; *p* < 0.01) and highest job requirement levels (highly complex: *β* = − 0.27; *p* < 0.001). For the total sample, the explained variance is 5%. Within the subsamples, ICT exposure explains 4% to 7% of variance.

#### Effects of ICT exposure on work ability

In the total study sample, ICT use showed a relevant positive effect on WA (*β* = 0.22; *p* < 0.001), but none on the other outcomes. Digital work intensification displayed a significant negative effect on WA (β = − 0.28; *p* < 0.001). When stratified, ICT use retained the significant effect on WA only in the specialist group (*β* = 0.20; *p* < 0.001). In contrast, the significant negative effect of digital work intensification on WA persisted in all job requirement levels (*β* = − 0.23 to − 0.31; unskilled/semi-skilled employees: *p* < 0.05, otherwise *p* < 0.001). For the total sample, the explained variance is 8.5%. The explained variance in the subgroups ranged from 4 to 8.9%.

## Discussion

This study provides a differentiated view on ICT exposure and its associations with health and work ability. Our findings show that work-related ICT use may be more common than expected even among older employees, with a prevalence of 92%. Interestingly, while one in five of overall workers did not experience any digital work intensification, an almost equally large proportion experienced high levels of digital work intensification. Both the proportions of workers reporting ICT use and digital work intensification increased significantly with an increase of SEP. This higher concentration of ICT exposure among workers with higher SEP was also shown in a large Swedish cohort study (Stadin et al. 2016).

Our results complement previous studies, which found that the introduction of new computer programmes at work partially moderates the association between working conditions and job dissatisfaction or psychosomatic health complaints, respectively (Meyer et al. 2019; see also Carayon 2007). In the present study, ICT use in and of itself did not appear to be negatively associated with the examined outcomes, which stands in contrast to descriptive findings from the German national employment survey suggesting that computer use is associated with worse mental health in the general population (BIBB/BAuA 2019). The different findings may, of course, be related to the fact that the national report’s findings are merely based on descriptive analyses. The only digital technology analysed was computer use and emotional exhaustion was measured as the mental health outcome. Furthermore, a relevant difference may be that in the present paper the effect of mere ICT use was adjusted for digital work intensification, the latter possibly being the stressing aspect of working with ICT.

Although some form of ICT use for work-related purposes was surprisingly widespread, the overall perception of digital work intensification was not particularly high in this sample of older workers. Digital work intensification is not associated with physical health, but showed overall negative associations with mental health and work ability. The magnitude of the negative effect of digital work intensification on mental health and WA was relatively low, but remained significant and similar across SEP. The strongest effects on mental health were in the lowest (unskilled/semi-skilled employees) and highest SEP (highly complex), however, all confidence intervals overlap. There is a consistently negative effect of digital work intensification on mental health and work ability also across all subsamples. All in all, when digital work intensification is perceived alongside ICT use, employees in each SEP appear to be negatively affected to a greater extent.

### The role of socioeconomic position

The stratification by SEP showed differences in proportion of ICT exposure. Even though the overall associations between ICT exposure and health outcomes in many cases were relatively weak, analyses by job requirement levels showed the risk of confounding effects (Hiesinger and Tophoven 2019). For instance, confounding appeared in positive associations of ICT use with work ability and physical health. That leads to the question whether the positive associations in the general sample are more or less a result of confounding by SEP: in higher SEP, positive health outcomes are to be expected more often than in lower SEP, and the same is true for ICT use. Future analyses should, therefore, generally integrate a variable, such as “job requirement” level, to control for SEP.

A recent large-scale study (Stadin et al. 2016) found a higher prevalence of ICT demands among participants with high and intermediate SEP applying a variable resembling the stratification in our study. The authors do not clearly distinguish whether the reported exposure to ICT-related stressors reflects different proportions of exposure to ICT. They also found associations of ICT demands with lower health outcomes to be similarly distributed across strata of SEP. The results of our study corroborate this finding with regard to SEP: the effects of digital work intensification on mental health show no significant difference in direction and strength in the lowest and highest job requirement levels. However, our results indicate that older employees’ ICT use is not associated with negative health outcomes per se. The extent to which personal resources and organisational factors may attenuate potential negative effects could be further explored (Lazarus 1966), specifically in the context of ICT use.

Our study aimed to take into account organisational factors and differences by SEP with regards to types of ICT use as ICT-related tasks. Further research and conceptual work on variables and stratification should aim to effectively differentiate between types of ICT use. For instance, whether a smartphone is used merely for work calls during working hours or whether it enables access to an application that functions as an automated management tool that controls work flows (Ivanova et al. 2018) may have very different implications for workers’ health.

### Strengths and limitations

A strength of the present study is that it draws on a large and representative sample of two cohorts (1959 and 1965) of employees in Germany and used well-validated measures of health and work ability outcomes (SF-12 and WA). Thus, the analyses were well-powered. Second, our study design based on personal interviews at the respondents’ homes allowed for the inclusion of respondents that may be averse to online surveys and avoid a bias towards people with a greater digital affinity (Khazaal et al. 2014). Our study complements previous research by providing a more comprehensive picture of respondents that cannot be reached online. Online surveys are particularly subject to coverage and selection bias, which can lead to unreliable survey outcomes and undermine the external validity of such studies (Bethlehem 2010). Third, this study controlled for relevant potential confounders, including not only age and gender, but also digital affinity beyond the workplace. Digital affinity may be important in the interplay of ICT exposure and health as it may be an indicator of how voluntarily people use ICT or how accustomed they are to certain technologies or the frequent use of ICT. It should be noted that other studies have included further confounders, such as Body Mass Index and health behaviour, yet we attempted to include confounders economically. To avoid overadjustment, every confounder should be well-justified before being included in analyses, but we could not find strong evidence for all the confounders used in previous studies.

To minimise methodological issues of many previous studies on ICT use and health at work (e.g. Stadin et al. 2016; Day et al. 2012; Ragu-Nathan et al. 2008), our choice of items sought to avoid conceptual overlap with negative health outcomes by choosing ICT exposure items with less negative connotations. Otherwise, we expect this would have artificially inflated correlations between ICT exposure and mental health or other stress scales. This issue regularly applies to studies using the technostress model, but other variables may also be affected. For instance, findings suggesting particularly high associations between ICT demands and the effort dimension of effort–reward imbalance may also be indicative of a relatively high propensity for conceptual overlap (Stadin et al. 2016, 2019). This may be due to the fact that the effort items specifically also ask about a high workload (e.g. “To what extent are you burdened because your workload increased over recent years?”). The question how to disentangle strain from different sources is indeed unresolved. Further studies focusing on that question should not only apply new ICT measures and traditional stress measures in parallel, but should develop a new framework where demands and resources as well as effort and rewards on the general and the ICT-level are operationalised in a complementary way. Thus, after considering the risks of conceptual overlap, we used items drawn from research on ICT demands measuring ICT exposures with a more neutral wording (Day et al. 2012).

However, the following limitations of the study should be addressed. First, the explained variance of the respective outcome variables is relatively small; therefore, conclusions from the data should be drawn with caution. But explaining a high share of variance cannot be the primary aim of a study like this since digital aspects of a working situation are only a limited part of working conditions. Additionally, alternative variables to measure psychosocial health or outcomes more directly related to work (e.g. job satisfaction) may be more sensitive than the more general physical and mental health. Second, due to the cross-sectional design, our study cannot provide information on the causal direction of the relationship between ICT exposure variables and health measures and no causal inferences can be drawn. Reversed causation cannot be excluded as mental health and work ability may lead to a perception of digital work intensification. Follow-up studies with longitudinal data from wave four of the lidA study (planned for 2022) will aim to overcome this limitation.

Third, due to the general limitations of surveys there is the problem of common method variance in self-reported data that may have led to inflation of associations. Fourth, the findings are technically only representative for older workers from two 1-year age cohorts (1959, 1965), which may represent the same generation in relation to their ICT use at work. In addition, participants were limited to workers subject to social security contributions, although the self-employed may be more or less prone to negative effects of ICT use and—depending on occupational group—may have a higher exposure. The high proportion of specialists (German: Fachkräfte) in the sample (55.2%) may be specific to vocational training in the German context and other countries may have different distributions; therefore, international studies are needed. Further studies could also aim to avoid the comparatively small size of the group of employees with unskilled or semi-skilled activities for greater statistical power in regression analyses, for example, through oversampling. Also, the healthy worker survivor effect should be kept in mind, meaning that workers with a high vulnerability to work stress and its consequences have lower chances of being in the sample (Chowdhury et al. 2017). A sample biased in this way may lead to an underestimation of the strengths of associations due to the restricted variance in the outcome.

## Conclusion

The results of this study point to the fact that ICT use in and of itself is not negatively associated with health and work ability. ICT use for work is relatively high among the study sample of older employees. But, unlike some previous research has suggested, our findings indicate that a high level of ICT use is not harmful when it is not experienced as digital work intensification.

Despite the broad exposure to ICT, older employees showed signs of negative outcomes only when ICT exposure included the experience of digital work intensification. Although digital work intensification did not appear to be a common experience, there may be specific and sizeable risk groups that account for the negative associations identified with mental health and work ability. As the effect sizes in our study are small, further contextual factors that influence the relationship need to be studied to better describe under what circumstances ICT use may have positive or negative health implications and whether they are modified by SEP. In contrast to previous research suggesting a stronger burden of ICT exposure with increasing SEP, our findings show that the negative associations with health implications are mostly of similar strength in all SEP. For instance, although a greater proportion of workers may be affected in higher socio-economic strata, the negative associations with mental health are of similar strength in the group with the lowest SEP and the negative associations with WA are similar across groups.

For practitioners in occupational health and organisational development, this means that it is important to monitor digitalisation processes to assess whether and to what extent they are implemented with the purpose of work intensification. At times, perhaps, digitally-induced work intensification may also be an unintended consequence. Moreover, attention should be given to all levels in the organisational hierarchy, in particular with regards to how digitalisation processes may entail unequal health and social implications.

### Implications for future research

Future studies could identify specific risk groups for whom the negative impacts of ICT exposure depend on whether digitalisation processes are implemented for purposes of work intensification. The typically stronger effect of digital work intensification in studies of occupational health and ICT (see e.g. Ragu-Nathan et al. 2008; Day et al. 2012) suggest that it may function as a proxy for a societal trend towards work intensification more generally. Depending on how one asks, respondents may attribute perceived issues not to ICT itself, but rather to the contextual factors that can mediate the impact of ICT use on work intensification: characteristics of the technology, goals of the organization, and an active role on the part of the users (Carayon 2007). Thus, more context-sensitive models of digital work and digitisation processes at the workplace are needed to better distinguish different forms of ICT use (Tarafdar et al. 2015). Such models could, for instance, aim to account for whether working with ICTs serves the purposes of algorithmic management (e.g. Apps used in gig economy food deliveries; see Ivanova et al. 2018).

While our study was interested in older employees, it could only analyse older employees as a general group and not as single cohorts. The age gap between the cohorts was too small to examine differences between them with regards to ICT exposure or the associations with health. Future studies should try to compare not only age groups that are more distant from each other, but also look into generational differences. It is to be expected that along with technological change over time, digital affinity, uses of ICT and their consequences change too.

Research indicating potential differences at the bottom and top of the social hierarchy warrants further investigation into whether digitalised processes of work intensification have implications for digital social inequalities (Halford and Savage 2010). Importantly, studies should consider to what extent models to operationalise ICT exposure and its associations with health can also account for differences by SEP. Despite being a fast-growing concern in occupational health research, the potential health-related outcomes linked to ICT use may still be conceptualised too narrowly. Health outcomes could also be examined together with work-related outcomes, such as job satisfaction and workplace well-being. Moreover, holistic, person-centred outcomes, such as fears due to surveillance or substitution at work, may facilitate capturing the ambiguous implications of digitalised work.

## Data Availability

The data used in this study are subject to strict federal data privacy legislation because the sample was drawn from social register data. Therefore, access for the public is prohibited. A Scientific Use File (SUF) containing data of the first two waves of the lidA study is available by request for scientific purposes: https://fdz.iab.de/de/FDZ_Individual_Data/lidA.aspx**,** and a similar file with be prepare for the data of wave 3.
